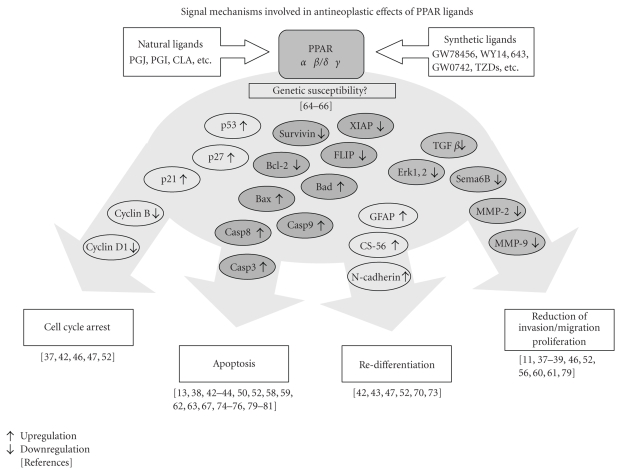# Peroxisome Proliferator-Activated Receptors (PPARs) as Potential Inducers of Antineoplastic Effects in CNS Tumors

**DOI:** 10.1155/2008/204514

**Published:** 2008-08-14

**Authors:** Lars Tatenhorst, Eric Hahnen, Michael T. Heneka

**Affiliations:** ^1^Department of Neurology, University of Bonn, Sigmund-Freud-Street 25, 53105 Bonn, Germany; ^2^Institute of Human Genetics, Institute of Genetics, and Center for Molecular Medicine Cologne (CMMC), University of Cologne, Kerpener street 34, 50931 Cologne, Germany

## Abstract

The peroxisome proliferator-activated receptors (PPARs) are ligand-inducible transcription factors which belong to the superfamily of nuclear hormone receptors. In recent years it turned out that natural as well as synthetic PPAR agonists exhibit profound antineoplastic as well as redifferentiation effects in tumors of the central nervous system (CNS). The molecular understanding of the underlying mechanisms is still emerging, with partially controverse findings reported by a number of studies dealing with the influence of PPARs on treatment of tumor cells in vitro. Remarkably, studies examining the effects of these drugs in vivo are just beginning to emerge. However, the agonists of PPARs, in particular the thiazolidinediones, seem to be promising candidates for new approaches in human CNS tumor therapy.

## 1. REVIEW CRITERIA

For this review we searched NCBI PubMed articles including early-release publications. Search terms included peroxisome proliferator-activated receptor (PPAR) in conjunction with “glioma” or “glioblastoma” or “astrocytoma” or “neuroblastoma.” The abstracts of retrieved citations were reviewed and prioritized by relevant content. Full articles were obtained and references were checked for additional material when appropriate. Only papers published in English between 1995 and 2008 were included.

## 2. PPARs

The peroxisome proliferators-activated receptors (PPARs) are ligand-inducible transcription factors which belong to the superfamily of phylogenetically related proteins termed nuclear hormone receptors (NHRs). Three different PPAR isotypes (PPAR*α*, PPAR*β*, also called *δ*, and PPAR*γ*) have been identified in various species and show structural homology [1, 2]. PPAR*γ* is found in two different isoforms, PPAR*γ*1 and PPAR*γ*2 [3].

PPAR*α*, PPAR*β*/*δ* and PPAR*γ* show unique spatio-temporal tissue-dependent patterns of expression during fetal development in a broad range of cell types with ectodermal, mesodermal, or endodermal origin. PPARs are involved in several aspects of tissue differentiation and development, such as the differentiation of the adipose tissue, brain, placenta, and skin [4]. Therefore, it appears that the PPAR isoforms developed from a common PPAR gene with broad ligand-binding specificity, itself derived from the ancestral orphan receptor [5].

PPARs regulate gene expression via multiple mechanisms, thereby functioning as obligate heterodimers with retinoid-X-receptors (RXRs). Like the other members of the NHR superfamily, PPARs are composed of four domains. The highly conserved DNA-binding domain together with its zinc finger domain is a common attribute of all family members. The DNA binding domain is linked to the C-terminal ligand binding domain by the hinge region. The E/F domain is responsible for the dimerization of PPARs with RXRs and the ligand-dependent transactivation function of the receptor. The N-terminal domain finally is involved in the ligand-independent regulation of the receptor activity (reviewed in [6]).

PPARs stimulate gene expression through binding to conserved DNA sequences, termed peroxisome-proliferator response elements (PPREs), present in the promoter region of their target genes. In the absence of ligands, these heterodimers are physically associated with corepressor complexes which suppress gene transcription [4]. However, upon binding of a ligand to the receptor, the NCor-containing corepressor complexes are dismissed and replaced with coactivator complexes. These coactivators are then linked to the basal transcriptional apparatus, thereby activating gene transcription [7].

PPARs act prinicipally as lipid sensors and regulate whole body metabolism in response to dietary lipid intake and direct their subsequent metabolism and storage [8]. The prototypic member of the family, PPAR*α*, was initially reported to be induced by peroxisome proliferators, and now denotes the subfamily of three related receptors. The natural ligands of these receptors are dietary lipids and their metabolites. The specific ligands interacting with the individual receptors have been difficult to establish, owing to the relatively low-affinity interactions and broad ligand specificity of the receptors.

PPAR*α* acts primarily to regulate energy homeostasis through its ability to stimulate the breakdown of fatty acids and cholesterol, driving gluconeogenesis and reduction in serum triglyceride levels. This receptor acts as a lipid sensor, binding fatty acids and intiating their subsequent metabolism. PPAR*α* binds a number of lipids including fatty acids, eicosanoids, and other natural lipid ligands. Its dominant action is to stimulate adipocyte differentiation and to direct lipid metabolites to be deposited in this tissue. PPAR*γ* operates at the critical metabolic intersection of lipid and carbohydrate metabolism. PPAR*γ* activation is linked to reduction in serum glucose levels, likely as a secondary effect of its ability to regulate endocrine factors. It is this latter activity that has led to the development of specific PPAR*γ* agonists for the treatment of type-2 diabetes [9]. The PPAR*β*/*δ* binds and responds to VLDL-derived fatty acids, eicosanoids including prostaglandin A1 [10] and appears to be primarily involved in fatty acid oxidation, particularly in muscle.

Binding of PPARs to their specific ligands leads to conformational changes which allow co-repressor release and co-activator recruitment. Even though all PPARs can be attributed to a common ancestral nuclear receptor, each PPAR isotype has its own properties with regard to ligand binding. Synthetic thiazolidinediones (TZDs), which are commonly prescribed for the treatment of type-2 diabetes, are selective PPAR*γ* ligands. Naturally occurring PPAR*γ* ligands include eicosanoids and the cyclopentenone prostaglandin 15d-PGJ2. The best characterized PPAR*γ* agonists are the TZDs including pioglitazone (Actos) and rosiglitazone (Avandia), which are Food and Drug Association (FDA) approved for treatment of type-2 diabetes. The TZD troglitazone (Rezulin) was introduced in the late 1990s but turned out to be associated with an idiosyncratic reaction leading to drug-induced hepatitis. It was withdrawn from the US market in 2000, and from other markets soon afterwards. There are a number of non-TZD-based PPAR*γ* agonists, such as GW78456 and others that have been developed. PPAR*α* ligands include fibrates that are commonly used for the treatment of hypertriglyceridemia and the synthetic agonists WY14,643 and GW7647. PPAR*β*/*δ* agonists include the prostacyclin PGI_2_, and synthetic agents including GW0742, GW501516, and GW7842. All three PPAR isotypes can be activated by polyunsaturated fatty acids with different affinities and efficiencies [8, 11]. An overview addressing the affinity of several natural and synthetic ligands has been summarized recently [12].

All PPARs have been described in the adult and developing brain as well as in the spinal cord. Furthermore, it has been suggested that PPAR activation in neurons may directly influence neuron cell viability and differentiation [13–17]. While PPAR*β*/*δ* has been found in neurons of numerous brain areas, PPAR*α* and *γ* have been localized to more restricted brain areas [18, 19]. The localization of PPARs has also been investigated in purified cultures of neural cells. PPAR*β*/*δ* is expressed in immature oligodendrocytes where its activation promotes differentiation, myelin maturation and turnover [20, 21]. The *γ* isotype is the dominant isoform in microglia. Astrocytes possess all three PPAR isotypes, although to different degrees depending on the brain area and animal age [22, 23].

The role of PPARs in the CNS is mainly related to lipid metabolism; however, these receptors have been implicated in neural cell differentiation and death as well as in inflammation and neurodegeneration. The expression of PPAR*γ* in the brain has been extensively studied in relation to inflammation and neurodegeneration [14]. PPAR*α* has been suggested to be involved in the acetylcholine metabolism [24] and to be related to excitatory amino acid neurotransmission and oxidative stress defense [18]. However, mice lacking PPAR*α* function appear healthy and fertile and do not show neurological phenotypes, suggesting that PPAR*α* is dispensable for brain development [25]. In contrast, loss of PPAR*γ* has been shown to be embyonically lethal [26]. Whereas PPAR*β*/*δ* remains highly expressed in the rat CNS, the expression of PPAR*α* and *γ* decreases postnatally in the brain [27]. In retina, all three receptors are expressed [23, 27, 28]. Even though this pattern of expression, which is isotype-specific and regulated during development, suggests that the PPARs may play a role during the formation of the CNS, their function in this tissue is still poorly understood. Both in vitro and in vivo observations show that PPAR*β*/*δ* is the prevalent isoform in the brain beeing found in all cell types, whereas PPAR*α* is expressed at very low levels predominantly in astrocytes [29]. Acyl-CoA synthetase 2, which is crucial in fatty acid utilization, is regulated by PPAR*β*/*δ* at the transcriptional level, providing a facile measure of PPAR*β*/*δ* action. This observation strongly suggests that PPAR*β*/*δ* participates in the regulation of lipid metabolism in the brain. This hypothesis is further supported by the observation that PPAR*β*/*δ* null mice exhibit an altered myelination of the corpus callosum. Such a defect was not observed in other regions of the central nervous system, and the expression of mRNA encoding proteins involved in the myelination process remained unchanged in the brain [30].

As mentioned above, PPARs were at first identified as controllers of lipid metabolism. Presently, it turned out that PPARs also play a role in controlling important cellular functions like energy homeostasis, diabetes, cell proliferation and cell death, differentiation, inflammation, and even cancer [6, 31]. Especially PPAR*γ* and its agonists have been demonstrated to induce antineoplastic effects in several types of cancer (reviewed in [7]). In the following we focus on the role of PPARs as potential inducers of antineoplastic effects in highly abundant CNS tumors, namely astroglioma and neuroblastoma.

## 3. ASTROGLIOMA

Malignant astrocytic gliomas represent the largest proportion of all primary brain tumors in adults [32, 33]. The characteristic feature of glioma cells is a high proliferation rate, accompanied by the ability to invade far into the healthy brain tissue. According to the WHO classification of tumors of the nervous system [32], gliomas are ranked with increasing malignancy in four classes from WHO grade I to WHO grade IV. The vast resistance against irradiation and chemotherapy and the prevalent recurrence after surgical resection are the main reasons for the poor prognosis in treatment of malignant astrocytic gliomas. Despite multimodal therapeutic approaches, the mean survival time of patients with WHO grade IV glioblastoma multiforme, which is also the most frequent brain tumor, is only about one year after diagnosis [33]. Although medical research has been intensified in the past decades, the overall survival of patients with malignant astrocytic gliomas was not essentially improved [34].

All isoforms of PPARs are expressed in the brain [35, 36] as well as in a variety of rat and human astroglial cell lines [7, 37–44]. PPAR*γ* has been shown to be expressed at high levels in human glioblastomas [31, 37, 45, 46]. Based on findings in other neoplastic disease, several natural and synthetic ligands of PPARs have been tested for their efficacy in the treatment of astroglioma. Bezafibrate and gemfibrozil, both PPAR*α* agonists, inhibited the cellular viability of glioblastoma cell lines [47]. A different effect was observed when human T98G glioblastoma cells were treated with other PPAR*α* ligands, clofibrate and Wy-14,643. These ligands strongly downregulated the expression of semaphorin 6B, a member of the semaphorin family of axon guidance molecules [39], suggesting suppression of glioma invasion mechanisms by these PPAR*α* agonists. However, no direct influence of Wy-14,643 on proliferation or induced cell death was observed in either human or rat glioma cells [43].

Treatment with conjugated linoleic acid (CLA) inhibited growth in primary human glioblastoma cells as well as ADF glioblastoma cells [13, 40, 48]. In ADF cells this was associated with an increase of PPAR*α* and a decrease of PPAR*β*/*δ* expression, whereas PPAR*γ* levels were unaltered [40]. Cimini et al. found that CLA and the PPAR*γ*-specific agonist GW347845 reduced glioma cell growth and induced apoptosis [13, 48]. The authors suggested that this effect was mediated by PPAR*γ* activation. This conclusion was supported by the finding that the PPAR*γ* antagonist GW259662 completely prevented both the CLA and GW347854X-induced effects on cell growth and apoptosis. Furthermore, PPAR*γ* agonists reduced cell adhesion, cell migration, and tumor invasion which was associated with a decrease in matrix metalloproteinase 2 (MMP2) levels. The authors stated that activation of PPAR*γ* is likely to be responsible for these latter effects, since the PPAR*γ* antagonist GW259662 completely abolished these effects [13]. Furthermore, treatment with CLA and GW347845 significantly decreased VEGF isoforms, indicating that PPAR*γ* may also inhibit angiogenesis in gliomas [48].

Pérez-Ortiz et al. reported that generation of reactive oxygen species (ROSs) was likely to be responsible for glitazone-induced glial cell death [35, 49], which is in line with findings of Kang et al. [50]. Interestingly, in four different glioma cell lines (A172, U87-MG, M059K, M059J) rosiglitazone led to inhibition of proliferation and induction of apoptosis in a PPAR*γ*-dependent way since there the antagonist GW9662 partially reverted this effect [46]. Ciglitazone and the putative natural PPAR*γ* ligand PGJ_2_ inhibited proliferation and induced apoptotic cell death in human [38] and rat glioma cells, and apoptotic cell death was correlated with the upregulation of Bax and Bad protein levels [43]. Similar effects have been described by Zang et al. [44], who also reported that a combination of pioglitazone with all-trans retinoic acid (ATRA) increased the cytotoxic effect. Tetradecylthioacetic acid (TTA), a saturated fatty acid and PPAR ligand, inhibited growth of BT4Cn rat glioma cells at increased levels as compared to the PPAR*γ* ligand rosiglitazone [37]. Furthermore, TTA reduced tumor growth and led to a longer survival of rats with implanted BT4Cn tumors. The use of the PPAR*γ* antagonist GW9662 reversed the effect of rosiglitazone but not for TTA, indicating that TTA might act both via PPAR*γ*-dependent and PPAR*γ*-independent pathways [37].

Grommes et al. reported that the nonthiazolidinedione tyrosine-based PPAR*γ* ligand GW7845 reduced viability of rat C6 and human glioma cells and induced apoptotic cell death in a PPAR*γ*-dependent mechanism as shown by the inhibition of these effects by the specific antagonist GW9662 [51]. Primary astrocytes were not affected, demonstrating the specificity of the effects of GW7845 on neoplastic cell types. GW7845 also reduced proliferation of rat C6 glioma cells and reduced both the migration and invasion of glioma cells [51]. These investigators have subsequently reported [52] that the PPAR*γ* agonist pioglitazone reduced cellular viability of rat and human glioma cell in vitro. Furthermore proliferation in rat glioma cells was inhibited, as measured by Ki-67 expression. Glioma cells overexpressing PPAR*γ*-cDNA showed reduced cellular viability after pioglitazone treatment, whereas treatment of glioma cells overexpressing a mutant cDNA lacking transcriptional activity, showed no antineoplastic effects [52]. Grommes et al. extended these findings to in vivo studies, using a C6 rat glioma model [52]. In this study, tumor volumes were dramatically reduced following pioglitazone administration intracerebrally, as well as orally, indicating that pioglitazone is able to cross the blood-brain barrier (BBB). It has not been established whether TZDs other than pioglitazone penetrate the BBB. However, in vitro studies provide evidence that troglitazone is actively incorporated by the bidirectional transporter Oatp14 (Slco1c1) expressed in brain capillary endothelial cells, which is likely to provide homeostasis of troglitazone and may be of other TZDs [53]. Treated animals showed drug-induced apoptosis in the tumors by activation of proapoptotic proteins. Grommes and coworkers also observed decreased tumor invasion in vivo which was correlated with reduced MMP9 levels. Indeed, PPAR*γ* agonists suppressed tumor migration in vitro in a Boyden chamber assay. Finally, they described a pioglitazone-induced upregulation of the astrocytic redifferentiation marker CS-56 in tumor cells both in vivo and in vitro. Primary astrocytes were not affected by pioglitazone, indicating the restriction of these effects to neoplastic cell types [52]. A possible explanation for this neoplastic specificity is given by Spagnolo et al., who showed differences in metabolic responses in GL261 glioma cells as compared to primary astrocytes when treated with the TZD troglitazone [54].

The same authors also presented a study exploring C57/Bl6 mice with an intracerebral glioma derived from GL261 cells [55]. Mice were treated with a combined therapy of interleukin (IL)-2-secreting syngeneic/allogeneic fibroblasts administered into the tumor bed along with the TZD pioglitazone. In contrast to the data of Grommes et al., only intracerebrally administered pioglitazone prolonged the survival of mice harboring an intracerebral glioma, whereas pioglitazone administered orally showed no effect. Finally, combination of pioglitazone and Il-2-secreting fibroblasts significantly prolonged the survival of the treated mice as compared to untreated animals [55].

Using an organotypic glioma invasion model, closely resembling extracellular matrix environment present in the brain, Coras et al. show that micromolar doses of troglitazone blocked glioma progression without neurotoxic damage to the organotypic neuronal environment observed [56]. The authors stated that the intriguing antiglioma property of troglitazone appears to be only partially based on its moderate cytostatic effects. Concordant with the data presented by Grommes et al., the authors showed that troglitazone effectively inhibits glioma cell migration and brain invasion. Interestingly, the antimigratory effects of troglitazone could be mimicked by inhibition of TGF-*ß* signaling which has shown to be intimately involved in glioma cell migration, suggesting both mechanisms to be interlinked. In this study, the authors identified troglitazone as a potent inhibitor of TGF-*ß* release, suggesting that troglitazone reduced glioma cell motility by counteracting TGF-*ß* signaling [56].

More than 10 years ago, Prasanna et al. [57] reported that treatment with lovastatin (a HMG-CoA reductase inhibitor) led to growth arrest in glioma cells, accompanied with an increased expression of PPAR. A combination therapy of lovastatin and the PPAR*γ* agonist troglitazone reduced cellular viability in the DBTRG-05MG human glioblastoma cell line [58]. Interestingly, the combination of lovastatin with two other PPAR*γ* agonists, rosiglitazone and ciglitazone, did not lead to the same effect. The authors suggested that it may be possible that PPAR*γ* is an essential, but not sufficient, factor in this synergism.

PPAR agonists have also been shown to exhibit effects on tumor biology through PPAR-independent mechanisms. For example, the PPAR*α*/*γ* dual agonist TZD 18 inhibited growth of T98G human glioblastoma cells and induced apoptosis through PPAR-independent mechanisms, since their respective antagonists MK-886 and GW9662 did not reverse this effect [59]. The TZD-mediated antineoplastic properties from PPAR*γ* was argued to arise from off-target, receptor-independent actions of the drugs as well as those of rosiglitazone and pioglitazone [35, 38, 43, 60]. The glitazones were toxic for the human glioma cell line U251 and rat glioma cell line C6, but not for primary rat astrocytes [43]. Indeed, PPAR*γ* seems not to be involved in these effects of the TZDs, since the inhibitor GW9662 had nearly no effect on attenuation of cytotoxicity. Using PPAR*γ* positive and PPAR*γ* deficient mouse embryonic stem (ES) cells, it has been demonstrated that the TZD troglitazone inhibited the growth of tumors formed by injection of PPAR*γ*+ and PPAR*γ*− cells to the same extent, indicating that PPAR*γ* is not essential for the antiproliferative effects of troglitazone [60]. Moreover, troglitazone derivatives which are unable to activate PPAR*γ* suppress cancer cell proliferation similar to troglitazone, giving further evidence that the antiproliferative effects of troglitazone are at least in part PPAR*γ*-independent [61]. Furthermore, troglitazone sensitized human glioma cells to TRAIL-induced apoptosis in a process independent of PPAR*γ* [62, 63]. Troglitazone treatment led to a marked downregulation of the antiapoptotic proteins FLIP and survivin [63] as well as Bcl-2 [62] and so could possibly counteract the capability of tumor cells to become resistant to apoptosis. Hence a combination therapy of troglitazone and TRAIL might be a promising experimental approach. Conversely, in A172 human glioma cells Kang and colleagues showed that the TZD ciglitazone induced cell death dependent of PPAR*γ*, but independent of caspase and AIF. Furthermore, the authors demonstrated that downregulation of XIAP and survivin is involved in the cell death mechanism [50]. A possible explanation for the differentiative effects of PPAR*γ* agonists was supposed to rely on PPAR*γ* dysfunction. Single strand conformational polymorphism (SSCP) analysis was carried out in different tumor and nontumor tissues, showing somatic loss-of-function mutations in different carcinomas [64, 65]. Genetic analysis of American patients with glioblastoma multiforme revealed an overrepresentation of the H449H polymorphism in the PPAR*γ* gene, possibly being an important low penetrance susceptibility locus for glioneural tumors [66].

## 4. NEUROBLASTOMA

Neuroblastoma is a phenotypically heterogeneous tumor, containing cells of neuronal, melanocytic or glial/Schwann cell lineage. Regardless of the phenotype, PPAR*γ* is expressed in neuroblastoma cell lines [67], in primary neuroblastoma cells [7] as well as in samples of patients harbouring neurblastoma [68]. Data about the expression of PPAR*β*/*δ* in neuroblastomas are scarce [69–71], and only a few studies report the expression of PPAR*α* at mRNA or protein level in human neuroblastoma cell lines [71–74]. Therefore, most studies that assess the influence of PPARs on treatment of neuroblastoma evaluate the impact of its natural or synthetic ligands.

The putative natural PPAR*γ* agonist 15d-PGJ_2_ inhibits cellular growth, decreases cellular viability and induces apoptosis in human neuroblastoma cells in vitro [67, 69, 74–76]. Rodway et al. [74] show that the PPAR*α* agonist WY-14643 has no effect on the growth of the IMR32 neuroblastoma cell line, whereas PGJ_2_ induces growth inhibition in the same neuroblastoma cells. This occurs through programmed cell death type II or autophagy, and the serum lysolipid LPA is responsible for modulating this cellular response. In the neuroblastoma cell line ND-7, the same group shows that the degree of PPAR*γ* activation induced by PGJ_2_ is modulated through an interaction with retinoblastoma protein (Rb) and the class I histone deacetylase 3 (HDAC3) [75]. A combination therapy consisting of PGJ_2_ and the histone deacetylase inhibitor trichostatin A (TSA) enhanced the growth inhibition effects and is therefore proposed as a promising new strategy in the treatment of neuroblastoma. It should be noted that the effects of 15d-PGJ2 can also arise from its actions on the NF*κ*B pathway [77]. Di Loreto et al. report that a specific PPAR*β* agonist as well as oleic acid induced redifferentiation in SH-SY5Y neuroblastoma cells [70].

The best studied synthetic PPAR*γ* agonists are the TZD class of antidiabetic drugs, also referred to as glitazones [7]. Valentiner et al. [78] tested four glitazones (ciglitazone, pioglitazone, troglitazone, rosiglitazone) and reported their in vitro effects on cell growth in seven human neuroblastoma cell lines (Kelly, LAN-1, LAN-5, LS, IMR-32, SK-N-SH, SH-SY5Y). All the glitazones inhibited cell growth and viability of the human neuroblastoma cell lines in a dose-dependent manner, whereas the effectiveness of the single drugs differed strongly between cell lines. Similar results for ciglitazone and rosiglitazone have been reported [75, 79]. Cellai et al. show that high concentrations of rosiglitazone significantly inhibit cell adhesion in vitro, invasiveness and apoptosis in SK-N-AS, but not in SH-SY5Y human neuroblastoma cells [79]. The authors argued that this effect may be related to cellular differences in PPAR*γ* transactivation. Furthermore, Jung et al. report that the TZD rosiglitazone protects SH-SY5Y cells against MPP+ as well as acetaldehyde-induced cytotoxicity, which may be ascribed to the induction of the expression of antioxidant enzymes and also to the regulation of Bcl-2 and Bax expression by rosiglitazone [80, 81].

## 5. CONCLUSION

The understanding of the molecular mechanisms underlying the antineoplastic effects mediated by PPAR agonists is still emerging. Over the past years, an increasing number of reports were published, presenting evidence for several involved pathways concerning cell cycle arrest, apoptosis, redifferentiation and inhibition of invasion/migration, that have been found to be affected by PPAR agonist treatment. Figure 1 presents an overview of signal mechanisms involved in the antineoplastic effects of PPAR ligands. Interestingly, there are partially controverse findings regarding the receptor dependency of the observed effects. Besides the number of natural and synthetic ligands, as well as to the number of different tumor cell lines used, a further explanation may be that most studies were performed on long-term cultured cell lines which may have undergone alterations while being in cell culture. Only few studies use primary cell cultures of tumor cells or organotypic models, like Benedetti et al. or Coras et al. [48, 56], trying to resemble natural conditions as close as possible. Remarkably, studies examining the effects of PPAR agonists in vivo are just emerging for gliomas [52, 55], and are still missing for neuroblastomas.

From all natural and synthetic PPAR ligands, the group of thiazolidinediones is the one with the best characterized antineoplastic properties. The fact that TZDs like pioglitazone (Actos) and rosiglitazone (Avandia) are FDA-approved for treatment of type-2 diabetes and therefore readily available for clinical studies may be the main reason for this. Very recently, a phase 2 clinical study was published, presenting for the first time a combination of low-dose chemotherapy with COX-2 inhibitors and PPAR*γ* agonists in high-grade gliomas [82]. Unfortunately, the trial had to be closed prematurely, due to the moderate efficacy as compared to other clinical trials, which however investigated PPAR*γ* agonist treatment of different tumor entities. It is questionable whether the tumor biology of astroglioma, which are extremely heterogeneous and rarely metastasize, can be compared to these different tumors, and thus the degree of response to a PPAR*γ* agonist-based therapy. Of note, depending on the particular astroglioma and region within the tumor, the poor blood brain barrier penetration of the TZDs may also account for limited efficacy. Therefore, further in vivo studies are warranted to unravel the molecular mechanisms underlying the antineoplastic effects of PPAR agonists in malignant astrocytic gliomas.

Nevertheless, agonists of PPARs, in particular the TZDs, seem to be promising candidates for new therapeutic approaches in human CNS tumor therapy due to their profound antiproliferative and anti-invasive effects as well as their positive effects on apoptosis and redifferentiation.

## Figures and Tables

**Figure 1:**